# Beating resonance patterns and extreme power flux skewing in anisotropic elastic plates

**DOI:** 10.1126/sciadv.adk6846

**Published:** 2023-12-20

**Authors:** Daniel A. Kiefer, Sylvain Mezil, Claire Prada

**Affiliations:** Institut Langevin, ESPCI Paris, Université PSL, CNRS, 75005 Paris, France.

## Abstract

Elastic waves in anisotropic media can exhibit a power flux that is not collinear with the wave vector. This has notable consequences for waves guided in a plate. Through laser-ultrasonic experiments, we evidence remarkable phenomena due to slow waves in a single-crystal silicon wafer. Waves exhibiting power flux orthogonal to their wave vector are identified. A pulsed line source that excites these waves reveals a wave packet radiated parallel to the line. Furthermore, there exist precisely eight plane waves with zero power flux. These so-called zero–group-velocity modes are oriented along the crystal’s principal axes. Time acts as a filter in the wave-vector domain that selects these modes. Thus, a point source leads to beating resonance patterns with moving nodal curves on the surface of the infinite plate. We observe this pattern as it emerges naturally after a pulsed excitation.

## Teaser

Laser-ultrasonic measurements reveal elastic waves propagating along the phase fronts and beating zero–group-velocity resonances.

## INTRODUCTION

Engineering materials often exhibit anisotropic stiffness, particularly single crystals and composites. Among them, monocrystalline silicon is the single most important material for fabrication of integrated circuits, microelectromechanical systems (MEMSs), and photovoltaic cells (*1*, *2*). These are generally produced from a thin wafer of this material. Understanding the intricate mechanics of elastic wave propagation in these plates is of importance not only for their quality evaluation (*3*–*5*) but also for the functional design of MEMS devices such as surface acoustic wave and Lamb wave filters and sensors (*6*–*8*). The latter usually involve layers of piezoelectric materials, which constitute another kind of medium where anisotropy plays a major role (*9*).

As the structures in the mentioned applications are usually thin, guided elastic waves are of great relevance. These waves propagate dispersively (*10*–*12*), i.e., their angular frequency ω is nonlinearly related to their wave number *k* through the dispersion relation ω(*k*). Of particular interest are solutions where the group-velocity component ∂ω*/∂k* vanishes while the wave number remains finite (*13*–*18*). In isotropic media, these zero–group-velocity (ZGV) points represent local resonances that are due to the finite thickness of the semi-infinite structure. At sufficiently high frequencies, they usually dominate the overall mechanical response of the structure. They are rather simple to excite and measure with contactless laser-ultrasonic techniques. ZGV resonances are used in nondestructive evaluation to determine various structural properties such as material parameters, thickness, or bonding state with very high precision in a spatially resolved manner (*19*–*23*).

**Fig. 1.**
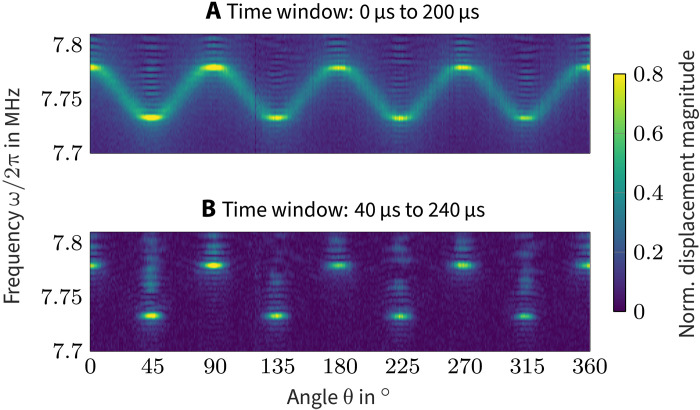
Angular dependence of the ZGV resonance. (**A** and **B**) The surface displacement was acquired at the center of a line source and obtained on two different time windows. The resonance frequency clearly depends on the line orientation with a 90° periodicity. θ is the angle of the excited wave vector and is counted from the [110] axis. For long times, the resonance remains at θ = *n* × 45°, *n* ∈ *ℤ*but vanishes elsewhere. The measurement data were taken from the experiment detailed in (*27*), where (A) has already been reported.

ZGV resonances in anisotropic plates were the object of some theoretical (*24*–*26*) and experimental studies (*27*, *28*). Prada *et al*. (*27*) revealed the directional dependence of the ZGV resonance frequency in a silicon wafer, and we reproduce this result in Fig. 1A (we will show, however, that not all of the components really correspond to ZGV resonances). Note that the directional dependence is distinctive of ZGV resonances as this is impossible for common thickness resonances characterized by *k* = 0. It is striking that the extremal frequencies in Fig. 1 play a special role, particularly when inspecting later times as depicted in Fig. 1B. Moreover, in (*27*), the response to the point-source excitation appears to be dominated by the two ZGV frequencies associated to the principal directions of the crystal. This fact was confirmed by numerical computations in (*25*). However, the described effects remain unexplained in the literature.

The aim of the present work is twofold. First, the special role of ZGV resonances along the principal directions of the material is explained. The contributions along other directions are explained by slow waves that propagate parallel to the line source, which means that they exhibit a group-velocity vector that is orthogonal to the wave vector. Second, we predict and confirm the formation of complex time-dependent resonance patterns on the surface of the plate after it has been excited by an impulsive point load. We show that this beating pattern can be explained by the interference of eight ZGV modes associated to the material’s principal directions.

## RESULTS

**Fig. 2.**
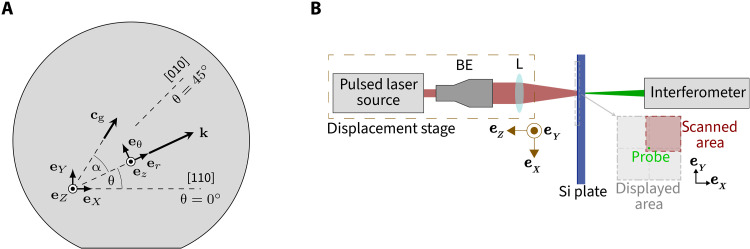
Sample and measurement setup. (**A**) [001]-cut silicon wafer sample. The **e***_X_***e***_Y_***e***_Z_* coordinate system is fixed as indicated, while the **e***_r_***e**_θ_**e***_z_* system is aligned with the wave propagation direction. The wave vector **k** is inclined at angle θ, while the group-velocity vector **c**_g_ is at angle θ + α. (**B**) schematic representation of the laser-ultrasonic measurement setup. BE, beam expander; L, lense.

We study transversely propagating waves and ZGV resonances in anisotropic plates. Both effects are confirmed experimentally on a [001]-cut monocrystalline silicon wafer of 524.6-μm thickness; see Fig. 2A. The material’s stiffness is of cubic anisotropy (Voigt-notated stiffness, *C*_11_ = 165.6 GPa, *C*_12_ = 63.9 GPa, and *C*_44_ = 79.5 GPa; density, ρ = 2330 kg*/*m^3^). The laser-ultrasonic system depicted in Fig. 2B is used to observe the waves. It consists of a pulsed laser source that is defocused to excite the wave-number range of interest of up to ≈7 rad/mm and of a laser interferometer that measures the normal surface displacements of the wafer. A two-dimensional (2D) scan of the surface is performed by moving the laser source. To speed up the measurement, we only scan one quarter of the wave field and reconstruct the full field by exploiting the sample’s material symmetries. Some of the results are band-pass–filtered between 7.6 and 7.8 MHz in a postprocessing step (indicated explicitly in the figures), as this frequency band contains the phenomena of interest. Further specificities on the measurement setup and the postprocessing can be found in the “Measurement setup” section. Before presenting the experimental results, the underlying theory is developed in the following.

### Guided waves in a silicon wafer

We study guided elastic waves propagating in the wafer. The fixed **e***_X_***e***_Y_***e***_Z_* coordinate system is aligned with the [110], [$\bar{1}10$], and [001] crystallographic axes, respectively. Moreover, the **e***_r_***e**_θ_**e***_z_* system is oriented with the wave vector, as depicted in Fig. 2A. Taking the point of view of a plane wave, **e***_r_* denotes the axial direction, while **e**_θ_ refers to the transverse direction. The plate has stress-free surfaces. Furthermore, it is considered to be of infinite lateral dimensions, so that waves reflected from the border can be disregarded.

Guided waves are characterized by the angular frequency ω, the wave vector **k** = *k***e***_r_*(θ) = *k_X_***e***_X_* + *k_Y_* **e***_Y_*, as well as the through-thickness displacement distribution **u**(*z*). Only certain combinations of frequency and wave vector can propagate, which is described by the dispersion relation (*10*–*12*, *29*). The solutions form surfaces ω(*k_X_*,*k_Y_*) or, equivalently, ω(*k*,θ). We use a semi-analytical method to obtain these solutions, i.e., the associated eigenvalue problem is solved numerically. Our implementation is made available as GEWtool (*30*). Therewith, we are also able to compute ZGV points (and the transversely propagating waves that will be discussed later on) by using the numerical methods that we have recently developed for this purpose (*24*, *31*). For details on the model and the numerical methods, see the “Computing guided waves and power flux” section.

**Fig. 3.**
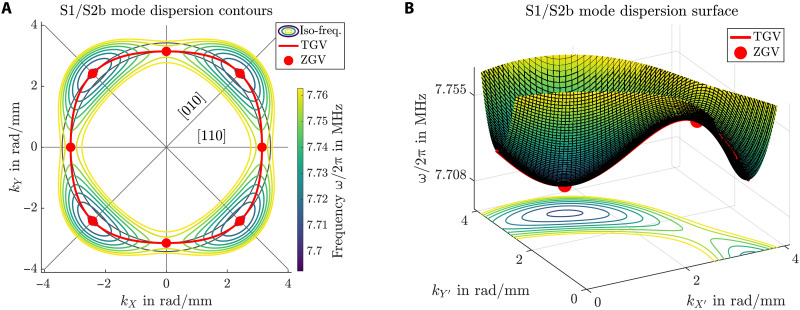
Dispersion surface of the S1/S2b mode close to the minima in frequency. (**A**) Iso-frequency contours in the wave-vector plane and (**B**) surface in the frequency-wave vector space. The ZGV points are located at the marked minima and saddle points (red points). For visualization purposes, (B) has been rotated by 22.5° and cropped to the shown quarter plane.

The computed dispersion surface of the first modes that exhibit ZGV points is shown in Fig. 3. Note that, because of the cubic anisotropy of silicon, the dispersion surface has an angular periodicity of 90°. Furthermore, we observe reflection symmetry across the directions θ = 0° and θ = 45°, which correspond to the [110] and [010] crystallographic axes, respectively. Furthermore, the surface exhibits four minima on the ⟨010⟩ axes and four saddle points along the ⟨110⟩ directions.

**Fig. 4.**
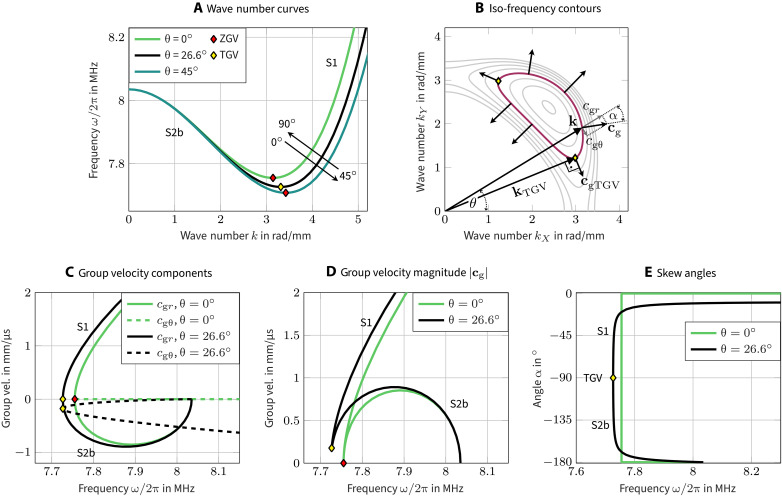
Dispersion of wave vectors and group velocity. (**A**) Frequency versus wave numbers. (**B**) Some group-velocity vectors sketched on a selected iso-frequency contour. Dispersion curves are cuts along constant θ of this surface. (**C**) Components *c*_g*r*_ and *c*_gθ_ of the group-velocity vector versus frequency, compare also to (B). The transverse power flux due to *c*_gθ_ does not vanish for TGV waves, while it does vanish at ZGV points. (**D**) Magnitude of the group-velocity vector versus frequency. (**E**) Skew angles α between the group-velocity vectors **c**_g_ and the wave vectors **k** plotted versus frequency.

Often, dispersion curves are plotted along a fixed propagation direction θ, corresponding to cuts across the dispersion surface ω(*k*,θ). The curves in three different propagation directions are depicted in Fig. 4A, and they differ most in the directions 0° and 45°. Although pure Lamb/shear-horizontal (SH) waves do not generally exist in the silicon plate because they are coupled, we label the mode branches consistently to the commonly used notation. In particular, following the notation in (*17*), the positive-slope branch shown in Fig. 4A will be denoted as S1, while the negative-slope section is called S2b.

The power flux of the waves is proportional to the group velocity (*10*–*12*, *32*). The latter is defined as the gradient of ω with respect to the components of the wave vector, i.e.

$$c_{g}:=\nabla_{k}\omega =\frac{\partial \omega }{\partial k}e_{r}+\frac{1}{k}\frac{\partial \omega }{\partial \theta }e_{\theta }$$ (1)

It is worth remarking that these vectors are, per definition, orthogonal to the iso-frequency lines drawn in Fig. 3A. This is illustrated in Fig. 4B, where some group-velocity vectors have been sketched for a chosen iso-frequency contour.

The power flux is discussed by comparing propagation at the principal direction θ = 0° and the nonprincipal direction θ = 26.6°. For both directions, we compute the group-velocity vectors as a function of frequency. The resulting vector components are depicted in Fig. 4C, while the magnitude is shown in Fig. 4D. Because of the material’s reflection symmetry across principal axes, the transverse power flux *c*_*g*θ_ is identically zero on the principal directions. This is not the case for other propagation directions, as Fig. 4C illustrates. Because of this, the corresponding group-velocity magnitude in Fig. 4D does no longer vanish at the minimal frequency. Furthermore, depending on whether the axial component *c*_g*r*_ is positive or negative, we speak of forward waves or backward waves (*17*, *33*–*35*), respectively. With respect to a source, a backward wave exhibits outgoing flux but incoming phase fronts. This is the case for the S2b wave. Note that the phenomena investigated hereinafter are strongly related to the existence of such waves.

The transverse-group-velocity (TGV) component *c*_gθ_ is due to the anisotropy ∂ω*/∂*θ and causes a transverse power flux. We remark that it is the coupling of Lamb and SH polarizations that implies this (usually) nonzero transverse power flux [appendix B of (*24*)]. Because of this transverse component, the overall power flux (or group velocity) is not collinear with the wave vector. The angle between the two is denoted as α and called steering or skew angle (*12*, *36*, *37*). The frequency-dependent skew angles of the S1/S2b modes are shown in Fig. 4E. It is remarkable that they cover almost 180° in a rather narrow frequency range.

### ZGV and TGV waves

Resonances appear where the power flux vanishes, and such points are denoted as ZGV points. This requires the vanishing of the two components of the group-velocity vector given in Eq. 1, i.e., these resonances are associated to stationary points of the dispersion surface of Fig. 3. It has been a common practice in the literature to plot the dispersion curves for a given propagation direction as in Fig. 4A to identify ZGV modes; see (*25*–*27*). However, these curves only reveal the axial component *c*_g*r*_ = ∂ω*/∂k* of the group-velocity vector. Thus, identifying ZGV points as extrema of these curves is only valid for isotropic media or for propagation along reflection symmetry planes of any anistroptic material, because, in these cases, the group-velocity vector is collinear to the wave vector, i.e., *c*_gθ_ ∼ ∂ω*/*∂θ ≡ 0. Hence, while the marked points on the curves of Fig. 4A appear to be ZGV points in the conventional sense that ∂ω*/∂k* = 0, their transverse power flux might actually be nonzero due to anisotropy. For this reason, we distinguish between.

1. ZGV points: waves with overall zero power flow, i.e., ∂ω/∂*k* = 0 and ∂ω/∂θ = 0, and

2. TGV waves: waves with zero axial power flux, i.e., ∂ω/∂*k* = 0.

A ZGV point is an extremum or saddle point of the dispersion surface depicted in Fig. 3. The cubic material exhibits eight isolated ZGV points on the principal directions. The four minima in the ⟨010⟩ directions are denoted as ZGV1 and occur at ω*/*2π = 7.7079 MHz and *k* = 3.421 rad/mm. The ZGV2 are saddle points at ⟨110⟩ with ω*/*2π = 7.7551 MHz and *k* = 3.142 rad/mm.

The existence of TGV waves is evident from the closed iso-frequency contours that enclose a minimum in Fig. 4B. These always exhibit points where the wave vector is tangent to the contour and, consequently, orthogonal to the group velocity. The wave number and frequency of TGV waves depend on the wave-vector orientation θ and form the continuous red curve drawn in Fig. 3 (A and B). The closed TGV curve separates the forward wave region (outside) from the backward wave region (inside).

#### TGV wave radiated by a line source

The existence of TGV waves is quite remarkable as their power flux is orthogonal to the wave vector. While this fact is obvious from their definition, the evolution of power flux when approaching the TGV frequency can be assessed in Fig. 4E: The skew angles of the S1 and S2b modes both converge to −90° when reaching the TGV point. Note that the weaker the coupling between Lamb and SH modes, the sharper we expect the transition toward −90° to take place. Ultimately, when these wave families decouple (i.e., on a principal direction or in an isotropic plate), we obtain a discontinuity as observed in Fig. 4E for θ = 0°. The combination of orthogonal propagation and wide skew-angle spectrum leads to extraordinary diffraction effects.

**Fig. 5.**
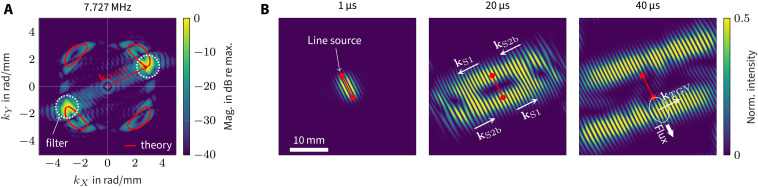
Measured TGV wave field radiated by a synthetic line source inclined 26.6°**. (**A**) Wave-number spectrum at the TGV frequency compared to theory prior to filtering in the wave-number domain. (**B**) Instantaneous intensity distributions after having applied a Gaussian filter in the wave-number domain as indicated in (A). The two wave packets propagate along the line source, while the wave vectors are orthogonal. Note that the phases propagate counter-wise in the two pulses, leading to interference where the two overlap. Frequency band from 7.6 to 7.8 MHz. Full field reconstructed from one quadrant (cf. movie S1).

To observe these effects, we synthesize a line-source response from the point-source measurements; for details, see the “Measurement setup” section. Waves usually radiate in normal direction from the line source and diffract around its edges. However, this is not the case for the TGV wave. This can be observed in Fig. 5, which presents the response to a 6.4-mm line source preferentially exciting wave vectors at 26.6° and −153.4°. The associated wave-number spectrum at the TGV frequency is compared to the theoretical wave numbers in Fig. 5A. To better observe the TGV wave, we apply a Gaussian filter in the wave-number plane. Its full width at half maximum is delineated in Fig. 5A. A subsequent inverse Fourier transform into the spatial domain yields the instantaneous intensity distributions depicted in Fig. 5B. Two wave packets propagating along the line source can clearly be discerned, i.e., their skew angle is ±90° as expected. From another point of view, this wave “diffracts” around the line’s edge while maintaining the orientation of the phase fronts aligned with the line source.

A further validation is done by comparing the group velocity of the wave packets to theory. After 40 μs, the maxima of the envelops are 6.81 mm from the source’s center, yielding a transverse velocity of ±0.170 mm/μs. This is in very good agreement with the computed value of −0.177 mm/μs for the TGV wave at θ = 26.6°, as shown in Fig. 4C. Note that the wave vectors oriented at θ = −153.4° lead to the wave packet traveling in the opposite direction.

Propagation of energy purely orthogonal to the wave vector is a strictly monochromatic process, both in frequency and wave number. The neighboring spectral components exhibit a nonzero axial power flux, and these waves are responsible for the packets’ lateral extend. Wave packets and their power flux can only be observed when considering a spectrum of finite width. This is ensured by the applied Gaussian filter in Fig. 5A, which has a half-width of 0.7 rad/mm. While all spectral components of the wave packets have very similar wave vectors, their power flux covers a wide range of directions. As the power flux is orthogonal to the iso-frequency lines, Fig. 5A shows that the skew-angle spectrum spans almost 180°.

Last, it is worth observing that the two wave packet’s phases propagate in opposite directions, as indicated in Fig. 5B. The counter-propagating wave packets lead to quickly varying interference in the regions where the two superpose. It is remarkable that the phase fronts of one packet travel all in the same direction. This means that the phases propagate toward the source on one side, and away from the source on the other side, as indicated by the wave-vector arrows in Fig. 5B. Accordingly, each wave packet clearly consists of the S1 forward wave and the S2b backward wave, separated spatially by the TGV component. This is consistent with the smaller wavelengths that we observe in Fig. 5B for the S1 components compared to the S2b components. The spatial allocation of modes is also in accordance with Fig. 4 (B and E).

#### Decay of the TGV wave contributions

The decay observed for some spectral components in the introductory Fig. 1 can now be explained. While the frequency extrema correspond to the ZGV resonances, the remaining spectral components are due to TGV waves. The line source excites the latter all along its length. The TGV waves then propagate along the source, where they are measured at the center point by the interferometer. Because of their propagative nature, the waves leave the detection point after a certain time. As seen in Fig. 1B, the TGV waves disappear completely after about 40 μs, while the expected eight ZGV resonances at θ = *n* × 45°, *n* ∈ ℤ, perdure.

The time for the TGV waves to escape the measurement point depends both on the length of the line source and the wave’s group velocity. Compared to other wave components, the TGV wave exhibits a very low group velocity, as can well be appreciated in Fig. 4D. Therefore, the TGV waves are detected for a relatively long time, resulting in a well-pronounced spectral peak in Fig. 1A. This explains why they play an important role in the presented measurements. Note that the group velocity tends to zero as the line orientation tends to one of the material’s principal axes. Hence, the closer the line orientation is to a ZGV point, the more pronounced the corresponding spectral peak will be.

### Resonance pattern of ZGV modes

The previous results demonstrate that time acts as a filter in the wave vector–frequency domain. After sufficient time, only the ZGV resonances remain in the finite spatial region of observation. As a consequence, a resonance pattern develops in the silicon crystal plate. We first lay out the theory explaining this resonance, and, subsequently, we present direct measurements thereof.

#### Theory of ZGV resonances

**Fig. 6.**
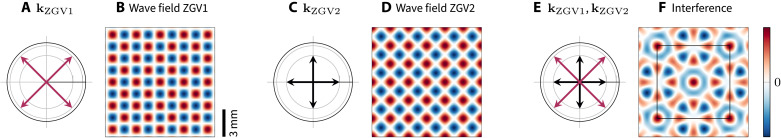
Formation of the S1/S2b resonance pattern in an infinite silicon plate. (**A**, **C**, and **E**) Wave vectors (not to scale). (**B**, **D**, and **F**) Corresponding wave fields. The ZGV modes in (A) and (B) and (C) and (D) are characterized by close but different wave numbers |**k**| and frequencies ω. These two standing wave fields interfere to form a nonstationary, time-dependent beating pattern as shown, for example, in (F) when the phase shift between the two is zero (cf. movie S2).

For the frequency region of interest, the resonance pattern is explained by the interference of the eight ZGV resonances sketched in Fig. 6 (A and C). The interference of the four wave vectors pertaining to a given ZGV mode leads to the periodic square wave pattern depicted in Fig. 6 (B and D). Note that, for each wave vector **k**, there exists a counter-propagating wave −**k**. As a consequence, the square patterns are standing wave fields (*17*). It is notable that this standing wave field is not due to the edges of the plate but actually emerges in the infinite plate.

It is pertinent to discuss differences between the isotropic and anisotropic cases. While the isotropic ZGV resonance consist of a continuous wave-vector spectrum, i.e., it exhibits wave vectors in all possible directions, the ZGV resonances in an anisotropic medium consist of a finite set of wave vectors. Accordingly, the ZGV field due to a point load on an isotropic plate is a cylindrical standing wave (*18*). In particular, this means that the nodal curves seen on the plate’s surface are closed circles around the source that never cross. In contrast to this, the field of the ZGV1 or ZGV2 resonance is the “checkerboard standing wave” as shown in Fig. 6 (B and D). Hence, anisotropy leads to straight, open, and crossing nodal lines.

The ZGV1 and ZGV2 waves interfere to form a resonance pattern as depicted in Fig. 6F. The nodal curves form “bubbles” of different shapes that are no longer square. As each of the wave components is flux free, no energy is propagated. Nonetheless, this is no longer a standing wave field because the ZGV1 and ZGV2 components are at different frequencies. Instead, the bubbles move radially out or into the center of the pattern while changing their shape (see movie S2). Note that they shortly stop moving when the two ZGV components are in phase or in paraphase. For the field synthesis shown in Fig. 6F, we arbitrarily assumed identical amplitudes and a zero phase shift between ZGV1 and ZGV2. Because of the similarity of the displacement eigenfunctions **u**_ZGV1_(*z*) and **u**_ZGV2_(*z*), both modes are excited similarly by the point source. For the same reason, we expect both ZGVs to be in phase at the moment of generation by the pulse.

The phase shift between ZGV1 and ZGV2 is modulated in time due to their slightly different frequencies. In other words, temporal beating is expected as a consequence of their superposition. Hence, the instantaneously observed phase shift loops 2π in a period of

$$\text{Δ}T=\frac{2\pi }{\omega_{\mathrm{ZGV}2}-\omega_{\mathrm{ZGV}1}}=21.2\;\mu s$$ (2)

#### Measurement of ZGV resonances

**Fig. 7.**
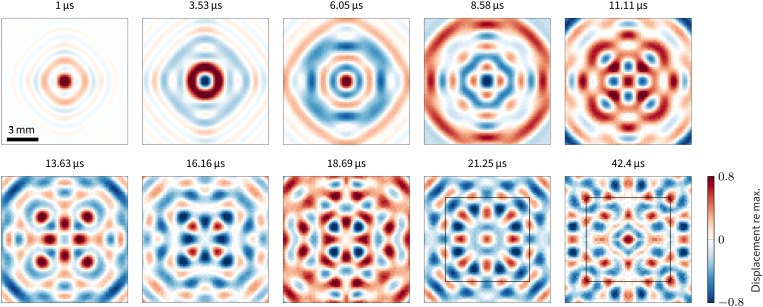
Measured wave fields at selected times. A resonance pattern forms due to the superposition of the eight plane waves corresponding to ZGV resonances in the single-crystal silicon wafer. The pattern forms naturally over time as only the ZGV resonances remain in the spatial window close to the point source. The inner region of 4 mm by 4 mm is marked for reference. Frequency band from 7.6 to 7.8 MHz. Full field reconstructed from one quadrant (cf. movies S3 and S4).

The described resonance pattern is directly observable by measuring the wave field excited by a point-like source. As the S1/S2b resonance is the one that is most strongly excited by our system, the theoretically expected moving resonance pattern can be discerned in the raw data without postprocessing; see movie S4. To obtain a clean result, other resonances as well as reflections from the border of the plate should be avoided. We achieve this with the mentioned frequency band-pass filter in combination with the large source that excites low wave numbers; see the “Measurement setup” section for details. The thus observed frequency–wave-number range contains only slow modes in the vicinity of the ZGV resonances. The filtered displacement fields are depicted in Fig. 7 for selected time instants.

Propagating waves dominate the picture at first, and their phase fronts (nodal curves) encircle the source. However, a nodal curve pattern with the mentioned bubbles forms over time; see Fig. 7 and movie S3. The pattern develops slowly from the source (in the center) outward as the propagative waves leave the region of excitation. When the pattern becomes first visible, the two ZGV resonances are not in phase and the bubbles move in and out on the ⟨110⟩ and ⟨100⟩ axes as expected from the theory.

After one beating period ∆*T*, the two ZGVs should be in-phase for the first time and, hence, we expect a pattern similar to Fig. 6F. At 21.25 μs, the pattern in the inner area marked by the square in Fig. 7 resembles the corresponding region in Fig. 6F. Outside this region, the contribution of propagating waves is still too high. Nonetheless, after two beating periods, at 42.4 μs, the non-ZGV waves propagated, and we can now recognize a fully developed ZGV resonance pattern in the region of observation. Note, for example, the “eight-pointed star” centered at the center and at the edges of the marked inner region. Discrepancies to Fig. 6F can mostly be attributed to temporal sampling of the measurement.

**Fig. 8.**
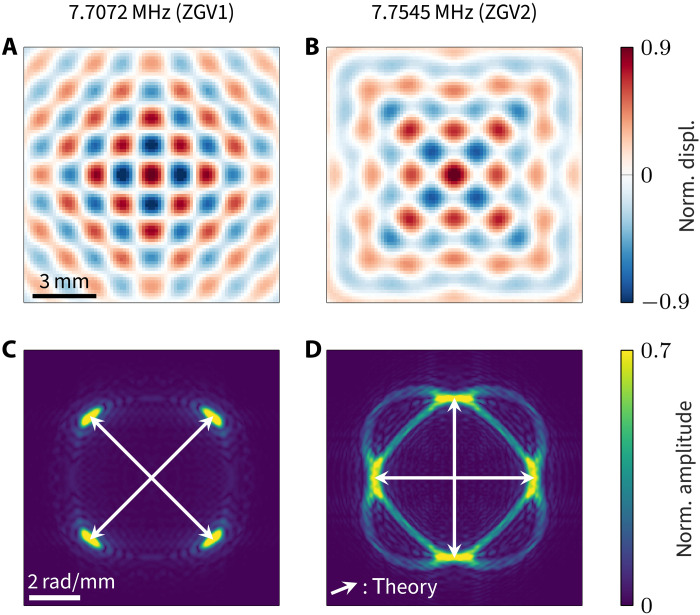
Measured displacement fields at the two ZGV frequencies. (**A** and **B**) Harmonic field at arbitrary phase in the physical *X*-*Y* space obtained by a temporal Fourier transform. (**C** and **D**) Spectral magnitude in the reciprocal *k_X_*-*k_Y_* space obtained by a spatiotemporal Fourier transform. The numerically computed ZGV wave vectors are indicated by arrows. Full field reconstructed from one quadrant.

A quantitative validation is performed by comparing measurement and theory in the spectral domain. First, a time Fourier transform yields the measured wave fields at the ZGV frequencies. The result is depicted in Fig. 8 (A and B), and both confirm the wave fields expected in Fig. 6 (B and D). Second, we additionally perform a spatial 2D Fourier transform into the wave vector–frequency domain. The obtained spectral amplitudes are displayed in Fig. 8 (C and D) together with the ZGV wave vectors expected from theory. We observe that the energy confines closely to the regions predicted by the computed wave vectors. While the wave field at ω_ZGV1_ consists almost purely of the ZGV resonances, propagating modes exist at ω_ZGV2_ and are also excited. This is because ω_ZGV2_ corresponds to the saddle points and the corresponding iso-frequency contours are two curves that cross/touch at the ZGV2 points. In contrast to this, the iso-frequency contours at ω_ZGV1_ reduce to the isolated ZGV1 points. Note that spatiotemporal gating can be used to remove the propagating waves of Fig. 8D, as was done in Fig. 1. Overall, this confirms that the resonances observed in an infinite plate of anisotropic elasticity are formed by the superposition of a discrete set of ZGV modes.

## DISCUSSION

The existence of isolated critical points in the S1/S2b mode dispersion surface entails two distinct and extraordinary effects: transverse wave propagation (TGV waves) and beating ZGV resonances. We presented the underlying theory and experimental evidence of both effects. TGV waves are characterized by a power flux that is orthogonal to their wave vector. They propagate along a line source that excites them. For this reason, they manifest as a peak in the spectrum measured at a chosen point of the line source.

ZGV resonances are already used very successfully for ultrasonic nondestructive testing and material characterization. With the developed physical understanding, we expect similar procedures to become attractive for novel MEMSs, particularly sensors. The present work overcomes one of the major difficulties in designing such systems: properly accounting for anisotropy, such as is encountered for silicon.

It is evident from the theory that we presented that the ZGV resonances directly reveal information about the principal axes of the material, independent of the actual orientation of the sample. Measuring the ZGV1 and ZGV2 resonance frequencies as well as the frequency of a regular thickness resonance is sufficient to fully characterize a material of cubic symmetry. These frequencies are readily obtained from the recorded normal surface displacement after a single pulsed excitation because they all manifest as sharp peaks in the spectrum (*27*). This avoids the technically complicated scan of the wave field, as needs to be done for widespread techniques based on guided waves. Overall, this could enable nondestructive, contactless, and real-time characterization of complex materials.

The present contribution restricted to ZGV resonances of the S1/S2b modes. However, it is well known that other ZGV modes exist at higher frequencies. They are substantially weaker and were frequency-filtered in this work. Note that anisotropic plates can also support multiple ZGV resonances pertaining to the same modes (*24*). In this case, not only local minima but also local maxima can appear in the dispersion surface. The analysis and mechanics presented here apply analogously.

The discussed phenomena are very particular to 2D (and partly also 3D) wave propagation in anisotropic media. Analogous phenomena should be expected when the material pertains to a different anisotropy class than the one studied here. Moreover, similar effects are expected in phononic crystals or meta-materials, as dispersion surfaces with similar features haven been reported for these materials (*38*–*41*). However, note that the critical points (ZGV points) that we studied here exist even without periodicity, i.e., in a homogeneous anisotropic plate. Last, hyperbolic polaritons share many of the wave propagation features described here, including power flux orthogonal to the wave vector (*42*).

## METHODS

The procedure that we use to compute guided elastic waves is sketched in the “Computing guided waves and power flux” section. The experiment is detailed in the “Measurement setup” section.

### Computing guided waves and power flux

The waves propagate in a plate that is assumed to be infinite in the *X*-*Y* plane; see Fig. 2A. Its material is of anisotropic stiffness **c** and mass density ρ. The plane waves of wave number *k* shall be harmonic with angular frequency ω. Hence, their wave field is of the form

$$\tilde{u}(r,\theta ,z,t)=u(z)\;e^{ikr}\;e^{-i\omega t}$$ (3)

We proceed by arbitrarily fixing the propagation vector **k** = *k***e***_r_* and computing the corresponding frequency ω and modal displacements **u**(*z*). These are obtained as the solutions of a differential eigenvalue problem (*24*, *29*, *43*). ω and **u**(*z*) represent the eigenvalues and the eigenfunctions, respectively. For a concise derivation of the concrete problem formulation, see (*24*).

We compute solutions with a semi-analytical procedure that consists in two steps: (i) discretize the differential eigenvalue problem and (ii) solve the resulting algebraic eigenvalue problem numerically. Many variants of this procedure have been discussed in the literature (*43*–*45*). A concise implementation based on spectral collocation is GEW dispersion script (*46*). For the current work, we perform the discretization using the *sp*ectral element method, i.e., one finite element of high polynomial order (*47*). This method leads to a regular Hermitian eigenvalue problem (*48*), which allows us to reliably compute the ZGV and TGV points in general elastic waveguides (*24*, *31*). We make our implementations available under the name of GEWtool (*30*), and it includes all required postprocessing methods to reproduce the results of this contribution. In particular, the script "dispersionSurface_silicon_ZGV.m" in the examples directory produces Fig. 3A.

The coupling and decoupling of wave families are of importance (*24*, *29*, *48*, *49*). Symmetric and antisymmetric waves decouple due to the plate’s symmetry across its midplane. We only compute the symmetric waves by modeling the top half of the plate and fixing the *u_z_* displacement component at the midplane. Moreover, the SH polarization (*u*_θ_) only decouples from the Lamb polarization (*u_r_*, *u_z_*) for θ = *n* × 45°, *n* ∈ ℤ. For this reason, we always compute the fully coupled waves, meaning that all three displacement components are accounted for in the displacement vector **u**(*z*).

After computing guided wave solutions, their power flux and group velocity can be computed in a postprocessing step. To this end, we exploit the fact that, in nondissipative waveguides (more precisely: real-valued **k** and ω), the group velocity is equal to the energy velocity (*10*, *32*, *50*) and is given by

$$c_{g}=c_{e}=\frac{\int p\;dz}{\int Hdz}$$ (4)

It is defined through the power flux density vector **p** and the average total stored energy density H. Using the particle velocity **v** = −iω**u** and stress **T** = **c **: ∇**u** = **c **: (i*k***e***_r_* + **e***_z_∂_z_*)**u**, the power flux density can be computed as

p=−12Re{v∗⋅T}=−12Re{iωu∗⋅c:(iker+ez∂z)u} (5)

where “${}^{*}$” denotes complex conjugation, “Re” the real-part operator, and ∂*_z_* = ∂/∂*z*. Furthermore, because of equipartition of energy (*12*), we can compute the average total stored energy density by

$$H=\frac{1}{2}\;{\rho \omega }^{2}u^{*}\cdot u$$ (6)

Our previously outlined procedure to solve for guided waves yields all quantities required to compute Eqs. 5 and 6. The differentiation in Eq. 5 and the integration in Eq. 4 can be performed numerically. In this way, we can compute the energy velocity of each point of the dispersion curves independently. Proceeding in this way explicitly provides both the axial and the transverse components of the energy velocity vector. The *z* component is identically zero due to the power flux–free surfaces of the plate. Last, as the wave vector is **k** = *k***e***_r_*, the skew angle α can be computed from the energy velocity vector as

$$\alpha =\left\{ \begin{array}{cc} \arctan\left(\frac{c_{e\theta }}{c_{er}}\right) & \mathrm{for}\;c_{er}\geq 0\\ \arctan\left(\frac{c_{e\theta }}{c_{er}}\right)-\pi & \;\mathrm{otherwise} \end{array}\right.$$ (7)

### Measurement setup

Measurements are achieved all-optically and are presented schematically in Fig. 2B. Guided waves are generated with a Q-switched Nd:YAG (yttrium aluminum garnet) laser (Quantel Laser, France, Centurion, 1064-nm optical wavelength, 100-Hz repetition rate) that delivers 10-ns pulses of 9 mJ. The laser output beam is expanded and then focused onto the silicon plate with a lens (100-mm focal length). The focal spot is kept rather large (beam width ≈ 1 mm), thus exciting wave numbers up to ≈7 rad/mm. This favors the generation of the first ZGV resonance while limiting the generation of the fundamental guided modes in the frequency range of interest (*51*). The wafer (525-μm nominal thickness, 524.6-μm measured thickness, 125-mm diameter) has a 100-nm aluminum coating on the excitation side, which reduces the optical penetration depth. However, similar results were obtained when exciting on the side without the coating. The coating is thin enough not to affect the elastic waves in the silicon plate.

The normal surface displacement is detected on the opposite side with a heterodyne interferometer of 532-nm optical wavelength with a power of 100 mW and a focal spot of ≈50 μm. A hardware high-pass filter with 1.25-MHz cutoff frequency is used to avoid saturation of the interferometer due to the large low-frequency displacements of the A0 mode. Signals are recorded with 100-MHz sampling rate by an oscilloscope connected to a computer. Each signal is averaged 128 times. The 25.05 mm–by–25.05 mm scan is performed by moving the excitation unit with a two-axis translation stage along **e***_X_* and **e***_Y_* with a 150-μm pitch. Note that scanning the field is time consuming. For this reason, we exploited the cubic symmetry of the material and scanned only one quadrant around the source, as indicated in the inset of Fig. 2B. The full fields have been reconstructed by an appropriate symmetrization.

While the measurements directly provide the point-source response, the line-source response shown in Fig. 5 is synthesized a posteriori. To this end, we superpose 20 shifted pointsource responses. The synthetic line source exciting wave vectors at θ = 26.6° is obtained by shifting two pitches vertically for every horizontal one.

Fast waves outside the ZGV region are also excited and their reflections from the border of the plate disturb the long-time observation of the slow TGV waves in Fig. 5 as well as the resonance patterns in Fig. 7. To avoid this, we use a band-pass filter between 7.6 and 7.8 MHz for these figures. Note that, because of the large source size, large wave numbers are not observed. The remaining frequency–wave-number range contains only the slow modes close to the ZGV points. 
