# Supplementary material for: Beating resonance patterns and extreme power flux skewing in anisotropic elastic plates
Source: Sci Adv. 2023 Dec 20;9(51):eadk6846. doi: 10.1126/sciadv.adk6846 (PMC10732515; doi:10.1126/sciadv.adk6846)
Supplement: Supplementary file 1 — Legends for movies S1 to S4 [file sciadv.adk6846_sm.pdf]

Science Advances
AAAS

# Supplementary Materials for

## Beating resonance patterns and extreme power flux skewing in anisotropic elastic plates

Daniel A. Kiefer *et al.*

Corresponding author: Daniel A. Kiefer, daniel.kiefer@espci.fr

*Sci. Adv.* **9**, eadk6846 (2023)
DOI: 10.1126/sciadv.adk6846

**The PDF file includes:**

Legends for movies S1 to S4

**Other Supplementary Material for this manuscript includes the following:**

Movies S1 to S4

## Description of supplementary videos

We provide four supplementary videos that are described in the following.

### Supplementary Video 1

Complement to Fig. 5: instantaneous intensity distribution at the surface of the plate. Two wave packets propagate along the line source while the wave vectors are orthogonal. Note that the phases propagate counter-wise in the two pulses, leading to interference where the two overlap.

### Supplementary Video 2

Complement to Fig. 6: theoretical wave field of the S1/S2b-resonance pattern at the surface of an infinite silicon plate. The ZGV1 and ZGV2 modes are characterized by close but different wavenumbers $k$ and frequencies $\omega$. These two standing wave fields interfere to form a non-stationary, time-dependent beating pattern shown on the right.

### Supplementary Video 3

Complement to Fig. 7: measured wave field after an impulse point source excitation at the surface of an infinite silicon plate (bandpass filter: 7.6 MHz–7.8 MHz). A resonance pattern forms due to the superposition of the eight plane waves corresponding to ZGV resonances. The pattern forms naturally over time as only the ZGV resonances remain in the spatial window close to the point source.

### Supplementary Video 4

Complement to Fig. 7: same as Supplementary Video 3 but without frequency-filtering. Except for reconstruction from the measured quadrant, no post-processing has been performed.
